# Cognitive subtypes in recent onset psychosis: distinct neurobiological fingerprints?

**DOI:** 10.1038/s41386-021-00963-1

**Published:** 2021-03-15

**Authors:** Julian Wenzel, Shalaila S. Haas, Dominic B. Dwyer, Anne Ruef, Oemer Faruk Oeztuerk, Linda A. Antonucci, Sebastian von Saldern, Carolina Bonivento, Marco Garzitto, Adele Ferro, Marco Paolini, Janusch Blautzik, Stefan Borgwardt, Paolo Brambilla, Eva Meisenzahl, Raimo K. R. Salokangas, Rachel Upthegrove, Stephen J. Wood, Joseph Kambeitz, Nikolaos Koutsouleris, Lana Kambeitz-Ilankovic, the PRONIA consortium, Mark Sen Dong, Anne Erkens, Eva Gussmann, Shalaila Haas, Alkomiet Hasan, Claudius Hoff, Ifrah Khanyaree, Aylin Melo, Susanna Muckenhuber-Sternbauer, Janis Kohler, Oemer Faruk Oeztuerk, David Popovic, Nora Penzel, Adrian Rangnick, Sebastian von Saldern, Rachele Sanfelici, Moritz Spangemacher, Ana Tupac, Maria Fernanda Urquijo, Johanna Weiske, Antonia Wosgien, Stephan Ruhrmann, Marlene Rosen, Linda Betz, Theresa Haidl, Karsten Blume, Mauro Seves, Nathalie Kaiser, Tanja Pilgram, Thorsten Lichtenstein, Christiane Woopen, Stefan Borgwardt, Christina Andreou, Laura Egloff, Fabienne Harrisberger, Claudia Lenz, Letizia Leanza, Amatya Mackintosh, Renata Smieskova, Erich Studerus, Anna Walter, Sonja Widmayer, Katharine Chisholm, Chris Day, Sian Lowri Griffiths, Mariam Iqbal, Paris Lalousis, Mirabel Pelton, Pavan Mallikarjun, Alexandra Stainton, Ashleigh Lin, Alexander Denissoff, Anu Ellila, R. N. Tiina From, Markus Heinimaa, Tuula Ilonen, Paivi Jalo, R. N. Heikki Laurikainen, Maarit Lehtinen, R. N. Antti Luutonen, Akseli Makela, Janina Paju, Henri Pesonen, Reetta-Liina Armio (Saila), Elina Sormunen, Anna Toivonen, Otto Turtonen, Ana Beatriz Solana, Manuela Abraham, Nicolas Hehn, Timo Schirmer, Carlo Altamura, Marika Belleri, Francesca Bottinelli, Marta Re, Emiliano Monzani, Mauro Percudani, Maurizio Sberna, Armando D’Agostino, Lorenzo Del Fabro, Villa San Benedetto Menni, Giampaolo Perna, Maria Nobile, Alessandra Alciati, Matteo Balestrieri, Giuseppe Cabras, Franco Fabbro, Sara Piccin, Alessandro Bertolino, Giuseppe Blasi, Linda A. Antonucci, Giulio Pergola, Grazia Caforio, Leonardo Faio, Tiziana Quarto, Barbara Gelao, Raffaella Romano, Ileana Andriola, Andrea Falsetti, Marina Barone, Roberta Passatiore, Marina Sangiuliano, Rebekka Lencer, Marian Surman, Olga Bienek, Georg Romer, Udo Dannlowski, Frauke Schultze-Lutter, Christian Schmidt-Kraepelin, Susanne Neufang, Alexandra Korda, Henrik Rohner

**Affiliations:** 1 University of Cologne, Faculty of Medicine and University Hospital of Cologne, Cologne, Germany; 2 Department of Psychiatry, Icahn School of Medicine at Mount Sinai, New York, NY USA; 3 Department of Psychiatry and Psychotherapy, Ludwig-Maximilian University, Munich, Germany; 4 International Max Planck Research School for Translational Psychiatry, Max Planck Society, Munich, Germany; 5 Department of Education, Psychology, Communication – University of Bari “Aldo Moro”, Bari, Italy; 6 Scientific Institute, IRCCS Eugenio Medea, San Vito al Tagliamento, Italy; 7 Department of Neurosciences and Mental Health, Fondazione IRCCS Ca’ Granda Ospedale Maggiore Policlinico, Milan, Italy; 8 Department of Pathophysiology and Transplantation, University of Milan, Milan, Italy; 9 Department of Radiology, University Hospital, Ludwig-Maximilian University, Munich, Germany; 10 Institute for Radiology and Nuclear Medicine St. Anna, Luzern, Switzerland; 11 Translational Psychiatry Unit (TPU), Department of Psychiatry and Psychotherapy, University of Luebeck, Luebeck, Germany; 12 Department of Psychiatry and Psychotherapy, Medical Faculty, Heinrich-Heine University, Düsseldorf, Germany; 13 Department of Psychiatry, University of Turku, Turku, Finland; 14 School of Psychology, University of Birmingham, Birmingham, UK; 15 Institute for Mental Health, University of Birmingham, Birmingham, UK; 16 Orygen, the National Centre of Excellence for Youth Mental Health, Melbourne, VIC Australia; 17 Centre for Youth Mental Health, University of Melbourne, Melbourne, VIC Australia; 18 Max Planck Institute for Psychiatry, Munich, Germany; 19 Institute of Psychiatry, Psychology and Neuroscience, King’s College London, London, United Kingdom; 20 Department for Psychiatry, Psychotherapy und Psychosomatics, University of Augsburg, Augsburg, Germany; 21 Max Planck School of Cognition, Stephanstrasse 1a, Leipzig, Germany; 22 Psychiatric University Hospital, University of Basel, Basel, Switzerland; 23 General Electric Global Research Inc, Munich, Germany; 24 Programma 2000, Niguarda Hospital, Milan, Italy; 25 San Paolo Hospital, Milan, Italy; 26 Department of Medical Area, University of Udine, Udine, Italy; 27 Department of Psychiatry and Psychotherapy, Westfaelische Wilhelms-University Muenster, Muenster, Germany

**Keywords:** Diagnostic markers, Psychosis, Cognitive neuroscience

## Abstract

In schizophrenia, neurocognitive subtypes can be distinguished based on cognitive performance and they are associated with neuroanatomical alterations. We investigated the existence of cognitive subtypes in shortly medicated recent onset psychosis patients, their underlying gray matter volume patterns and clinical characteristics. We used a K-means algorithm to cluster 108 psychosis patients from the multi-site EU PRONIA (Prognostic tools for early psychosis management) study based on cognitive performance and validated the solution independently (*N* = 53). Cognitive subgroups and healthy controls (HC; *n* = 195) were classified based on gray matter volume (GMV) using Support Vector Machine classification. A cognitively spared (*N* = 67) and impaired (*N* = 41) subgroup were revealed and partially independently validated (*N*_spared_ = 40, *N*_impaired_ = 13). Impaired patients showed significantly increased negative symptomatology (*p*_fdr_ = 0.003), reduced cognitive performance (*p*_fdr_ < 0.001) and general functioning (*p*_fdr_ < 0.035) in comparison to spared patients. Neurocognitive deficits of the impaired subgroup persist in both discovery and validation sample across several domains, including verbal memory and processing speed. A GMV pattern (balanced accuracy = 60.1%, *p* = 0.01) separating impaired patients from HC revealed increases and decreases across several fronto-temporal-parietal brain areas, including basal ganglia and cerebellum. Cognitive and functional disturbances alongside brain morphological changes in the impaired subgroup are consistent with a neurodevelopmental origin of psychosis. Our findings emphasize the relevance of tailored intervention early in the course of psychosis for patients suffering from the likely stronger neurodevelopmental character of the disease.

## Introduction

In accordance with the neurodevelopmental hypothesis [1] the majority of patients suffering from psychosis show general and specific neurocognitive impairments [2, 3] as premorbid signs of early developmental insults and brain alterations [4]. However, studies report substantial heterogeneity regarding the severity of neurocognitive impairments [2] putatively representing different underlying disease trajectories marked by specific (neuro-)biological, clinical and functional characteristics [5].

Impaired cognitive and psychosocial functioning represent the top of the dysfunctional pyramid of schizophrenia (SZ) [6]. For a number of patients with psychosis, cognitive impairment persists beyond the presence of positive and negative symptoms and relates to reduced psychosocial outcome [6]. For this reason, identifying homogeneous subgroups of patients showing specific cognitive profiles may enhance the effects of promising novel treatments including neurocognitive interventions [7]. Previous studies using unsupervised machine learning (ML) found between two and four cognitive subgroups in SZ samples, ranging from unimpaired to severely deteriorated patient subgroups [8–11]. These subgroups differed not only with respect to their cognitive performance yet also in clinical symptomatology [8, 9, 11], general [8, 10, 11] and occupational functioning [9, 11]. Furthermore, they were linked to different patterns of alterations in brain morphology [10, 12]. Complementary, studies using unsupervised ML identified neuroanatomical subgroups that were related to differences in premorbid functioning [13, 14] and neuropsychological performance [14].

Existing evidence on cognitive subgroups is mainly based on chronic SZ samples presenting with clinical symptoms for a prolonged period. These findings could be limited as patients may already be susceptible to change due to the effects of antipsychotic medication on cognitive performance [15] and brain structure [16].

The current study aims at disentangling variability in neurocognitive impairment. To achieve this, we (1) subgroup a recent onset psychosis (ROP) sample based on neurocognitive performance using cluster analysis and validate the cluster solution on neurocognitive data of an independent validation sample [17], (2) associate obtained ROP subgroups to symptom burden and functional disability and (3) investigate morphological brain differences between the cognitive subgroups and healthy controls (HC) using gray matter volume (GMV) within a supervised ML framework.

## Materials and methods

### Sample

In the discovery sample 121 ROP patients and 201 HC, age between 15 and 40 years, were recruited within the PRONIA study (Personalized Prognostic tools for early psychosis management; www.pronia.eu; German Clinical Trials Register: DRKS00005042) at seven sites across Europe. Patients were included in the study if they fulfilled DSM-IV-TR criteria [18] for a psychotic episode present in the last 3 months, lasting longer than 1 week and with first onset in the last 24 months [19]. HC volunteers were required to not fulfill any current or past DSM-IV-TR axis I or II diagnosis, clinical high-risk (CHR) status for psychosis as defined by the Structured Interview for Prodromal Syndromes [20] and Schizophrenia Proneness Instrument [21] or positive familial history (1st degree relatives) for psychosis accompanied by a drop in functioning in the last year. HC participants with any intake of psychotropic medications more than five times/year or in the month before study entry were excluded. Written informed consent was obtained from the subjects. The study received ethical approval by each Local Research Ethics Committee at every study site separately (Supplementary Materials and Methods) [19].

The independent validation sample comprised baseline data of a monocentric, longitudinal cognitive intervention study called Personalized Neurocognitive Training (ClinicalTrials.gov Identifier: NCT03962426). Overall, 58 ROP patients were recruited at the Early Detection and Intervention Center at the Department of Psychiatry and Psychotherapy of the Ludwig-Maximilians-University in Munich, Germany. Inclusion and exclusion criteria were identical to those required for the discovery sample of the PRONIA study.

The analysis data set consisted of 108 ROP patients and 195 HC for the discovery sample and 53 ROP patients for the independent validation sample (Table 1, Fig. S8, Supplementary Materials and Methods).

**Table 1:** Demographic and clinical characteristics of the discovery and validation sample used in the study.

	Discovery				Validation			Validation vs. discovery	
			ROP vs. HC			ROP vs. HC		ROP (val) vs. ROP (disc)	
	ROP (*N* = 108)	HC (*N* = 195)	*t/X²*	*p*	ROP (*N* = 53)	*t/X²*	*p*	*t*	*p*
Demographics									
Age	24.91 (5.11)	25.32 (6.23)	−0.63	0.53	25.74 (6.39)	0.42	0.68	0.82	0.41
Site^a^	39/20/28/8/13	48/39/60/35/13	11.62	0.02*	53/0/0/0/0	98.1	<0.001***	59.26	<0.001***
Sex^a^	Female = 35	Female = 121	23.28	<0.001***	Female = 21	7.67	0.01*	0.53	0.47
Years of education	14.08 (3.3)	16.02 (3.43)	−4.83	<0.001***	14.05 (3.54)	−3.62	<0.001***	−0.06	0.96
Illness duration in days	181.51 (187.46)	–	–	–	186.38 (203.88)	–	–	−0.15	0.88
Chlorpromazine equivalent^b^	388.18 (1020.61)	–	–	–	1208.09 (5205.17)			−1.06	0.29
Premorbid intelligence									
WAIS (Vocabulary)	9.89 (3.64)	12.11 (2.85)	−5.48	<0.001***	9.22 (3.3)	−5.61	<0.001***	−1.13	0.26
WAIS (Matrices)	9.35 (2.7)	11.23 (2.25)	−6.14	<0.001***	10.35 (2.73)	−2.15	0.03*	2.16	0.03*
GAF (symptoms)									
Lifetime	77.77 (10.09)	88.48 (5.63)	−10.15	<0.001***	77.22 (8.79)	−8.61	<0.001***	−0.35	0.73
Past year	59.12 (15.79)	87.43 (6.1)	−17.83	<0.001***	62.3 (14.19)	−12.24	<0.001***	1.26	0.21
Past month	41.85 (13.52)	86.98 (6.48)	−32.54	<0.001***	39.86 (13.02)	−24.81	<0.001***	−0.88	0.38
GAF (disability)									
Lifetime	77.11 (8.99)	86.84 (5.21)	−10.29	<0.001***	75.78 (9.74)	−7.75	<0.001***	−0.82	0.42
Past year	61.36 (13.66)	85.95 (5.82)	−17.76	<0.001***	61.82 (14.21)	−11.76	<0.001***	0.19	0.85
Past month	45.39 (12.24)	85.51 (6.16)	−31.78	<0.001***	42.8 (11.77)	−24.8	<0.001***	−1.27	0.21
PANSS									
Positive scale	18.07 (6.43)	–	–	–	20.27 (4.72)	–	–	−2.39	0.02*
Negative scale	16.75 (8.11)	–	–	–	15.33 (6.21)	–	–	1.2	0.23
General scale	36.05 (10.6)	–	–	–	34.02 (10.02)	–	–	1.15	0.25
BDI score	20.91 (11.41)	2.80 (4.73)	−14.91	<0.001***	22.44 (12.79)	−10.14	<0.001***	−0.69	0.49

*ROP* recent onset psychosis, *HC* healthy control, *WAIS Wechsler Adult Intelligence Scale, GAF* General Assessment of Functioning, *PANSS* Positive and Negative Syndrome Scale, *BDI* Beck Depression Inventory.

^a^Chi-squared test.

^b^Cumulative sum of Chlorpromazine equivalents divided by number of days treated.

*p < 0.05, ***p < 0.001.

### Clinical and neurocognitive assessment

Participants were assessed using multiple clinical scales and neuropsychological tests focusing on the General Assessment of Functioning Scale (GAF) [22], split into two subscales (symptoms and disability), the Global Functioning Scale (GF social and occupational) [23] and the Positive and Negative Syndrome Scale (PANSS) [24]. The neuropsychological test battery comprised of ten tests that were assigned to cognitive domains comparable to the MATRICS Consensus Cognitive Battery (MCCB) domains [25] including visual memory (Rey–Osterrieth Complex Figure test [26]), social cognition (Diagnostic Analysis of Non-Verbal Accuracy [27]), working memory (Auditory Digit Span Task [28], Self-ordered Pointing Task [29]), processing speed (Verbal Fluency Test [30], Trail Making Test A [31], Digit-Symbol-Substitution Test [28]), verbal learning and memory (Rey Auditory Verbal Learning Test [32]), executive functioning (Trail Making Test B [31]), attention and vigilance (Continuous Performance Test, Identical Pairs version [33]) and one psychosis-specific domain: aberrant salience [34] (Tables S1, S2 and Supplementary Materials and Methods).

### Preprocessing and clustering of neurocognitive data

All selected neurocognitive variables were used. Preprocessing followed the steps of (1) imputing missing values by median and (2) linear regression of effects of age, sex, years of education and study site to account for site and demographic differences [35]. In addition, we used (3) principal component analyses (PCA) for dimensionality reduction on each group of neuropsychological variables associated with a certain cognitive domain (Table S1) and retained the first PCA component of each domain for cluster analysis (Fig. S1).

A K-means clustering algorithm [36] was applied to the neurocognitive domain values (PCA components) using Euclidean distance. Two independent resampling strategies were followed to assess cluster stability [37].

Preprocessing of the validation sample followed procedures identical to the discovery sample. To estimate the generalizability of the discovery clustering model to new observations, cluster assignment in the validation data set was based on the minimum Euclidean distance of a single observation to the centroids of the discovery sample cluster solution.

Demographic, clinical and neuropsychological characteristics of the obtained ROP subgroups and the HC sample were compared using one-way permutation and chi-squared tests. *P* values were corrected using the Benjamini–Hochberg false discovery rate method [38] (Supplementary Materials and Methods).

Preprocessing, clustering and statistical analyses were conducted in R version 3.6.1 (https://cran.r-project.org/bin/windows/base/). Cluster stability was assessed using the ‘clusterboot’-function [37] contained in the ‘fpc’ package [39]. Cluster assignments of the validation observations were predicted using the ‘flexclust’ package [40]. Characteristics of subgroups were compared using non-parametric statistical tests from the ‘coin’- and the ‘rcompanion’-package [41, 42].

### Preprocessing of neuroimaging data

MRI data were inspected for scanner artefacts and anatomical abnormalities by a trained radiologist. Images were preprocessed using the open-source CAT12 toolbox (version > r1200; http://dbm.neuro.uni-jena.de/cat12/), an extension of the SPM12 software (Wellcome Department of Cognitive Neurology, London, UK; http://www.fil.ion.ucl.ac.uk/spm/software/spm12/) following previously described steps [19] and the CAT12 manual (www.neuro.uni-jena.de/cat12/CAT12-Manual.pdf) (Supplementary Materials and Methods).

### Neuroimaging classification analysis

A ML pipeline was employed to compare GMV between the obtained clusters and the HC population. Model generation and testing were embedded in a tenfold × tenfold nested cross-validation pipeline with ten permutations on inner (CV1) and outer (CV2) loop using the in-house ML tool NeuroMiner (http://www.pronia.eu/neurominer) running in MATLAB 2019a (MathWorks Inc.).

Within CV1 modulated, normalized GMV images were (1) smoothed with a Gaussian kernel (optimized for 4, 6 and 8 mm), (2) corrected for total intracranial volume and (3) pruned by removing zero-variance voxels. Moreover, images were (4) pruned for voxels with low reliability across study sites using a G coefficient map to account for scanner differences [19], (5) dimensionality was reduced by PCA (optimizing the retainment of the highest ranking components optimizing 40, 60 and 80%) and (6) values were scaled between zero and one.

To find a discriminative pattern of GMV between groups, a linear support vector machine (SVM) algorithm (optimized c-parameter range between 0.015625 and 16; 11 parameters) weighted by group sizes was applied on the GMV maps. Model performance was assessed by calculating the balanced accuracy (BAC). Statistical significance of the overall winning model was assessed using permutation tests (*N*_perm_ = 1000; alpha = 0.05) [43]. Reliability of discriminative voxels contributing to the classification performance of the winning model was inspected by the cross-validation ratio (Supplementary Materials and Methods).

## Results

### Discovery sample

A two-cluster solution indicated maxima on the Calinski-Harabasz index [44] and the average silhouette width score [45]. Stability assessment revealed clusterwise Jaccard similarity [46] indices of 0.84 and 0.90 for the ‘subset’ and 0.90 and 0.93 for the ‘noise’-method, respectively, indicating highly stable clusters (Fig. S3) [37].

### Neurocognitive characteristics

Patients in cluster 1 (*N* = 41) showed significantly lower performance in processing speed (*p*_fdr_ < 0.001, *d* = 1.89), executive functioning (*p*_fdr_ < 0.001, *d* = −1.60), attention (*p*_fdr_ < 0.001, *d* = 1.01), working memory (*p*_fdr_ = 0.004, *d* = 0.67), verbal (*p*_fdr_ < 0.001, *d* = −1.37) and visual memory (*p*_fdr_ < 0.001, *d* = 1.44) as compared to patients belonging to cluster 2 (*N* = 67).

Cluster 1 patients showed significantly lower performance in processing speed (*p*_fdr_ < 0.001, *d* = 2.11), executive functioning (*p*_fdr_ < 0.001, *d* = −0.77), attention (*p*_fdr_ < 0.001, *d* = 1.01), working memory (*p*_fdr_ < 0.001, *d* = 1.10) and verbal (*p*_fdr_ < 0.001, *d* = −2.43) and visual memory (*p*_fdr_ < 0.001, *d* = 1.66) as compared to HC group. We refer to cluster 1 as ‘impaired’ due to its largely inferior cognitive performance in comparison to cluster 2 and HC.

**Fig. 1:**
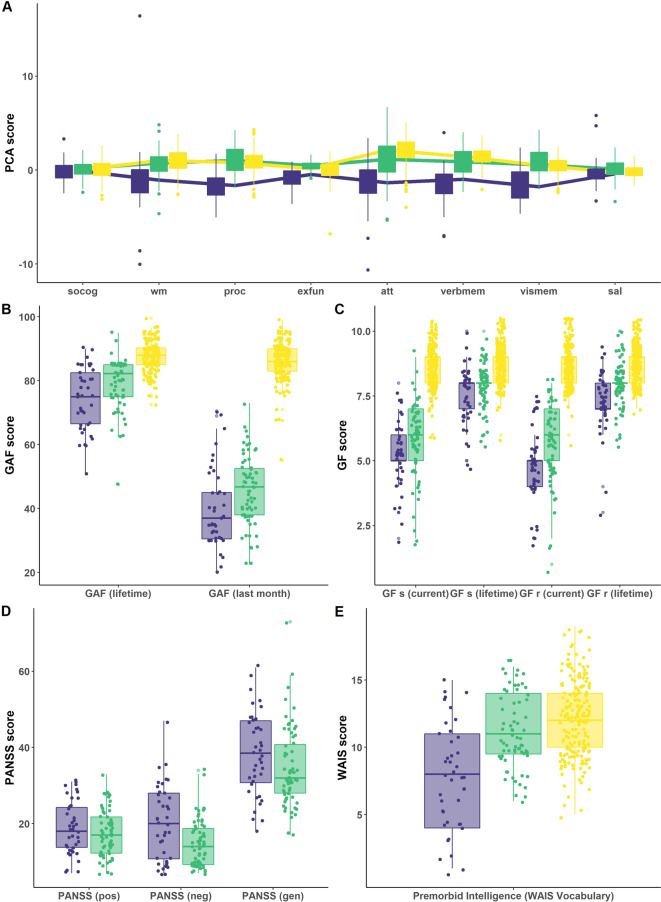
Neuropsychological and clinical differences between clusters and HC in the discovery sample. Differences between the impaired (blue; *N* = 41) and spared cluster (green; *N* = 67) and HC (yellow; *N* = 195) regarding **A** the neuropsychological PCA components, **B** the General Assessment of Functioning score (GAF), **C** the General Functioning score (GF), **D** the Positive and Negative Syndrom Scale (PANSS) and **E** Premorbid Verbal Intelligence are shown. **A** High PCA scores represent high performance. PCA scales for cognitive domains where high PCA scores represent low performance, are inverted. socog social cognition, wm working memory, proc processing speed, exfun executive functioning, att attention, verbmem verbal memory, vismem visual memory, sal salience.

Cluster 2 patients showed significantly decreased performance in attention (*p*_fdr_ < 0.001, *d* = 0.65) and verbal memory (*p*_fdr_ = 0.001, *d* = −0.47) as compared to HC. They showed improved performance in executive functioning (*p*_fdr_ < 0.001, *d* = 0.53), salience (*p*_fdr_ = 0.003, *d* = 0.44) and visual memory (*p*_fdr_ = 0.003, *d* = 0.44) compared to HC. We refer to this cluster as ‘spared’ as its performance was inferior to HC only in two cognitive domains (Table 2 and Fig. 1A).

**Table 2:** Neuropsychological domain-specific effects between impaired and spared cluster and healthy controls in discovery and validation sample.

	Overall			Impaired vs. spared		Impaired vs. HC		Spared vs. HC	
	*T* (max)	*p* (uncorr)	*p* (FDR)	*p* (FDR)	*Cohen’s d*	*p* (FDR)	*Cohen’s d*	*p* (FDR)	*Cohen’s d*
Discovery									
Social cognition	0.980	0.583	0.583	–	–	–	–	–	–
Working memory	6.089	<0.001	<0.001***	0.004**	0.68	<0.001***	1.11	0.053	0.28
Processing speed	10.070	<0.001	<0.001***	<0.001***	1.90	<0.001***	2.12	0.223	−0.17
Executive functioning	5.416	<0.001	<0.001***	<0.001***	−1.62	<0.001***	−0.78	<0.001***	0.53
Attention	8.756	<0.001	<0.001***	<0.001***	1.02	<0.001***	2.05	<0.001***	0.65
Verbal memory	10.385	<0.001	<0.001***	<0.001***	−1.39	<0.001***	−2.44	0.001**	−0.48
Visual memory	8.423	<0.001	<0.001***	<0.001***	1.45	<0.001***	1.67	0.003**	−0.44
Salience	2.646	0.022	0.023*	0.175	−0.28	0.913	−0.02	0.003**	0.45
Validation									
Social_cognition	2.824	0.012	0.014*	0.008**	−1.13	0.010*	−0.75	0.159	–
Working_memory	0.792	0.700	0.720	–	–	–	–	–	–
Processing_speed	7.256	<0.001	<0.001***	<0.001***	1.91	<0.001***	2.48	0.007**	0.50
Executive_functioning	2.497	0.031	0.034*	0.020*	0.98	0.023*	0.67	0.212	–
Attention	0.249	0.965	0.965	–	–	–	–	–	–
Verbal_memory	7.112	<0.001	<0.001***	<0.001***	−1.48	<0.001***	−2.51	0.050	–
Visual_memory	8.628	<0.001	<0.001***	<0.001***	2.29	<0.001***	3.04	0.052	–
Salience	3.533	0.001	0.001**	0.008**	−1.12	<0.001***	−1.03	0.578	–

*HC* healthy control.

*p < 0.05, **p < 0.01, ***p < 0.001.

### Demographic characteristics

Cognitively impaired patients showed significantly reduced number of years of education (*p*_fdr_ < 0.001) and a significantly decreased female-to-male ratio (*p*_fdr_ = 0.009) compared to HC. Patients in the spared cluster showed significantly lower number of years of education (*p*_fdr_ = 0.002) and lower female-to-male ratio (*p*_fdr_ < 0.001) as compared to HC. The number of patients recruited across sites differed significantly for the two clusters (*p*_fdr_ = 0.046) and when comparing the impaired group and HC (*p*_fdr_ = 0.014). Clusters did not differ regarding chlorpromazine equivalent level (*p*_fdr_ < 0.100) and illness duration (*p*_fdr_ < 0.440) (Table 3).

**Table 3:** Demographical effects between *impaired* and *spared* cluster and healthy controls in discovery and validation sample.

	Impaired	Spared	Overall			Impaired vs. spared	Impaired vs. HC	Spared vs. HC
	Mean (sd)	Mean (sd)	*T*(max)/*Z*	*p* (uncorr)	*p* (FDR)	*p* (FDR)	*p* (FDR)	*p* (FDR)
Discovery								
*N*	41	67						
Age	23.5 (4.3)	25.8 (5.4)	2.015	0.106	0.109	–	–	–
Years of Education	13.5 (3.2)	14.5 (3.3)	4.612	<0.001	<0.001***	0.135	<0.001***	0.002**
Sex^a^	female = 16	female = 19	25.611	<0.001	<0.001***	0.302	0.009**	<0.001***
Site^a,b^	11/5/11/5/9	28/15/17/3/4	23.614	0.003	0.003**	0.046*	0.061	0.014*
Ìllness duration in days^c^	163.66 (153.82)	192.43 (205.69)	−0.770	0.440	0.440	–	–	–
Chlorpromazine equivalent^d^	685.65 (1596.42)	196.95 (125.38)	1.940	0.052	0.100	–	–	–
Validation								
N	13	40						
Age	24.2 (5.3)	26.2 (6.7)	0.899	0.630	0.673	–	–	–
Years of Education	14.3 (3.7)	14.0 (3.5)	3.594	<0.001	0.001**	0.858	0.081	0.001**
Sex^a^	Female = 5	Female = 16	8.575	0.014	0.016*	1.000	0.140	0.017*
Illness duration in days^c^	149.00 (91.46)	198.53 (228.55)	−0.761	0.447	0.535	–	–	–
Chlorpromazine equivalent^d^	127.80 (267.83)	1578.48 (6006.66)	−0.833	0.405	0.535	–	–	–

*HC* healthy control, *sd* standard deviation, *FDR* False Discovery Rate.

^a^Nominal permutation test are used; Fisher’s exact *p* value is reported.

^b^Sites: Munich/Basel/Köln/Udine/Milan.

^c^Difference in time between first fulfillment of psychotic diagnosis according to Structured Clinical Interview for DSM-IV (SCID) and date of MRI examination.

^d^Cumulative sum of chlorpromazine equivalents divided by the number of days treated.

*p < 0.05, **p < 0.01, ***p < 0.001.

### Clinical characteristics

Cognitively impaired patients showed significantly lower premorbid intelligence (*p*_fdr_ < 0.001, *d* > 1.04), lower GAF score in the last month (*p*_fdr_ = 0.027, *d* = 0.49), in the last year (*p*_fd_ = 0.035, *d* = 0.46) and lifetime (*p*_fdr_ = 0.011, *d* = 0.59) and lower GF scores at examination (*p*_fdr_ < 0.045, *d* > 0.43), last year (*p*_fdr_ < 0.50, *d* > 0.42) and across lifetime (*p*_fdr_ < 0.024, *d* > 0.51) when compared to patients in the spared cluster. Cognitively impaired patients showed significantly higher scores on the PANSS negative scale (*p*_fdr_ = 0.003, *d* = −0.72) (Table S4 and Fig. 1B–E).

### Validation sample

Observations in the validation sample were assigned to the impaired (impaired_val_, *N* = 13) and spared (spared_val_; *N* = 40) cluster of the discovery sample.

### Neurocognitive characteristics

Cognitively impaired_val_ patients showed significantly worse performance in social cognition (*p*_fdr_ = 0.008, *d* = −1.13), processing speed (*p*_fdr_ < 0.001, *d* = 1.91), executive functioning (*p*_fdr_ = 0.020, *d* = 0.98), salience (*p*_fdr_ = 0.008, *d* = −1.12) and verbal (*p*_fdr_ < 0.001, *d* = −1.48) and visual memory (*p*_fdr_ < 0.001, *d* = −2.29) compared to cognitively spared_val_ patients.

Cognitively impaired_val_ patients performed significantly worse regarding social cognition (*p*_fdr_ = 0.010, *d* = −0.75), processing speed (*p*_fdr_ < 0.001, *d* = 2.48), executive functioning (*p*_fdr_ = 0.023, *d* = 0.67), salience (*p*_fdr_ < 0.001, *d* = −1.03) and verbal (*p*_fdr_ < 0.001, *d* = −2.51) and visual memory (*p*_fdr_ < 0.001, *d* = 3.04) when compared to HC.

Cognitively spared_val_ patients showed significantly reduced performance in processing speed (*p*_fdr_ = 0.007, *d* = 0.50) in comparison to HC.

### Demographic characteristics

Cognitively impaired_val_ patients showed no significant differences to cognitively spared_val_ patients and HC. Cognitively spared_val_ patients showed a significantly lower number of years of education (*p*_fdr_ = 0.001) and lower female-to-male ratio (*p*_fdr_ = 0.017) compared to HC. Clusters did not differ regarding chlorpromazine equivalent level (*p*_fdr_ = 0.535) and illness duration (*p*_fdr_ = 0.535) (Table 3).

### Clinical characteristics

Cognitively impaired_val_ patients showed significantly lower premorbid intelligence (*p*_fdr_ < 0.001, *d* = 1.66) and lower GF scores for role functioning last year (*p*_fdr_ = 0.042, *d* = 0.87) and across life span (*p*_fdr_ = 0.042, *d* = 0.87) when compared to cognitively spared_val_ patients (Table S4 and Fig. S5B–E).

### sMRI classification results

**Fig. 2:**
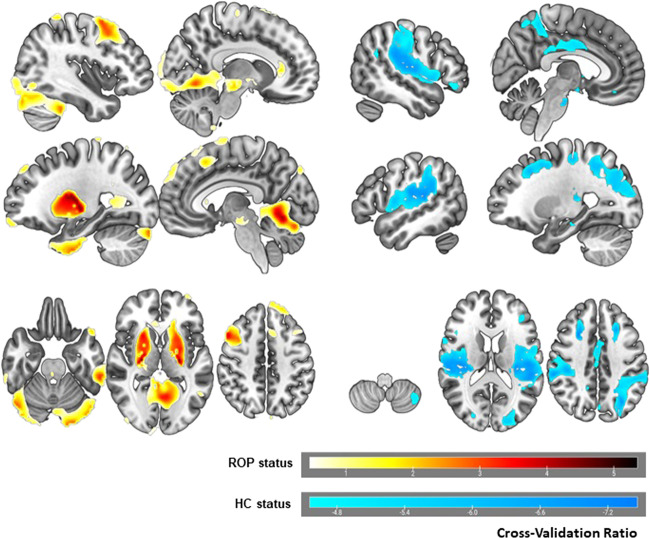
Reliability of predictive voxels for the impaired vs. HC classification model. Voxel-wise reliabilities are represented by the cross-validation ratio. Warm colors represent the 10% most reliable voxels predicting impaired ROP status, i.e., areas with increased gray matter (GM) in ROP. Cool colors represent the 10% most reliable voxels predicting HC status, i.e., areas with increased GM in HC. Left and right hemisphere are reversed.

A neuroanatomical SVM classification model discriminated the cognitively impaired patient group from HC (BAC = 60.1%, sensitivity = 56.1%, specificity = 64.1%, NND = 5.0; *p* = 0.01) in the discovery sample. The classification model of the cognitively spared group against the HC (BAC = 55.4%, sensitivity = 47.8%, specificity = 63.1%; *p* = 0.09) and the cognitively spared group against the cognitively impaired group (BAC = 47.2%, sensitivity = 31.7%, specificity = 62.7%; *p* = 0.79) remained non-significant (Fig. 2).

The neuroanatomical signature between cognitively impaired ROP and HC group comprised both cortical and subcortical regions. Bilateral GMV increases associated with ‘cognitively impaired ROP’ status were predominantly found in basal ganglia and cerebellum and to a lesser extent in the middle frontal and inferior temporal gyrus. The unilateral GMV decreases were localized in the right superior frontal, supplementary motor areas and anterior cingulum. Left lateralized reductions were found in inferior occipital and orbito-frontal gyrus and superior temporal pole.

Increases in GMV associated with HC status were found bilaterally in the Heschl’s gyrus, supramarginal gyrus, superior temporal gyrus and rolandic operculum. Further, bilateral increases in GMV were located in superior frontal and middle occipital regions, precuneus, in the cingulum and parahippocampal gyrus. The unilateral GMV increases were shown in left inferior frontal areas and cerebellum alongside with GMV increases in right superior parietal regions and angular gyrus, inferior orbital gyrus and hippocampus.

## Discussion

Our study reveals two cognitively and clinically distinct neurocognitive subgroups in ROP patients in line with previously reported cognitive subgroups in chronic SZ patients [8–11]. To the best of our knowledge, this is the first study showing altered cognitive, clinical and neuroanatomical features, using unsupervised ML methods, in the early stages of psychosis when patients are minimally affected by antipsychotic medication. We obtain a largely impaired and a spared subgroup and validate both in an independent behavioral data set of ROP patients. Whilst the applied neuroanatomical classification analysis was successful in distinguishing the cognitively and clinically impaired cluster from HC, it revealed no statistical differences between the spared subgroup and HC.

The current study found an impaired cluster presenting with more profound cognitive deficits in the domains of processing speed, working memory, executive functioning, attention and visual and verbal memory in comparison to HC. The spared cluster shows impairments in attention and verbal memory relative to HC, however, a similar performance in working memory, processing speed and social cognition. Conversely, this cluster shows increased performance in executive functioning, salience and visual memory relative to HC (Fig. 1 and Table 2). Increased performance in a psychosis subgroup relative to HC has been reported in a previous study [47]. The presence of cognitively and functionally preserved individuals in one subgroup might have been easier to identify due to our minimally medicated recent onset sample in comparison to previously employed chronic patient cohorts [8–11].

Analysis of the cognitive clusters’ clinical characteristics revealed premorbid general functioning [8, 10, 11], social and occupational functioning [9, 11] difficulties in the impaired group which were less present in the spared group (Supplementary Table S4). In line with prior studies, we confirmed a higher level of negative symptoms in impaired ROP patients as compared to the spared ROP patients [8, 9] (Supplementary Table S4). Importantly, though making a major contribution to the cluster solution, cognitive subgroups were not entirely explained by premorbid intelligence (Supplementary Materials and Methods).

Similar as in the discovery sample, we found reduced performance in processing speed, executive functioning and verbal and visual memory alongside impaired premorbid intelligence level and partially impaired functioning for impaired_val_ patients when compared to spared_val_ patients and HC of the independent behavioral data set. The concordance on verbal memory and processing speed deficits between impaired patients across both samples supports recent efforts of the second phase of the North American Psychosis Longitudinal Study-II that generated a risk calculator for transition to psychosis integrating both domains in its prediction model [48].

Our classification analysis reliably showed patterns of GMV increases associated with impaired-cluster status predominantly in the subcortical area of putamen [13] while we observed smaller increases in cortical areas [49]. Basal ganglia enlargement seems to occur in medication-naive populations with clinical and genetic risk [50]. As our ROP patients were newly exposed to antipsychotic treatment, larger basal ganglia appear to reflect striatal hyperdopaminergia possibly related to acute psychotic symptoms [51]. In previous studies, unaffected family members have also shown larger putamen [51]. However, HC have shown increases in fronto-temporo-parietal cortical regions with an emphasis on Heschl’s gyrus [52] and parahypocampal areas [53] which are particularly prone to GMV loss in psychosis [16, 49].

Previous studies propose a preadolescent decline trajectory for SZ, characterized by impaired premorbid intelligence, reduced general cognition at illness onset and lower level of occupational functioning [11]. First, impaired patients show high levels of negative symptoms [8, 9] and gradual differences in social and occupational functioning in comparison to spared subgroup and HC. Second, studies demonstrate developmental lags relative to same-aged HC [54] in CHR individuals who go on to develop full-blown psychosis. Large cohort studies in CHR [55] implicate that immediate verbal learning, memory and processing speed are the most relevant domains for prediction of transition to psychosis. Those domains are significantly reduced in our impaired subgroup (Supplementary Fig. S9) and replicate in the validation sample. Third, previous cross-sectional findings on ultra-high risk (UHR) individuals who later transitioned to psychosis reported reduced GMV in prefrontal areas, temporal gyrus and cerebellum relative to HC and to UHR who did not transition to psychosis, respectively [56, 57]. In the current study, the impaired subgroup shows a significant neuroanatomical signature relative to HC. The presence of GMV reduction, despite the absence of chronicity and long-term medication effects, suggests these brain alterations may have emerged before the onset of florid psychotic symptoms. Finally, both behavioral and imaging effects persist after controlling for differences across subgroups regarding age, sex, educational years, study site and group sizes. In addition, post hoc examination of the relationship between decision scores of the ‘impaired subgroup vs HC’ neuroimaging classification model and study site ensures that our classification model is not mainly driven by site-specific scanner differences (Supplementary Materials and Methods).

The current study has several limitations. First, the applied neuropsychological tasks differed from the MCCB [25] and cognitive domains, e.g., social cognition and executive functioning, were underrepresented in comparison to other tests (Table S1). Second, we could only partially replicate the effects of the discovery cluster solution. This might be due to differences in sample characteristics and sizes (Table 1) or the monocentric characteristic of the validation sample. Third, while we suggest that the characteristics of the impaired subgroup align with early maladaptive processes as proposed in the neurodevelopmental hypothesis [1], our assessment of functioning is retrospective and cross-sectional. Future studies would benefit from a longitudinal design providing a more comprehensive answer. Fourth, as cross-site data acquisition differences arise as key issues in multi-center studies [58], we accounted for such effects in both behavioral and neuroimaging analysis. However, an effect of an unbalanced distribution of participants between subgroups and HC on our cluster findings cannot be ruled out entirely.

Cognitive and clinical differences in the psychosis subgroups of the discovery sample support the idea of distinct trajectories in early stages of the disease [5]. In accordance with this finding is the neurobiological separability of cognitively impaired patients from HC. Early detection of psychosis subgroups could help to tailor early interventions for ROP patients with likely stronger neurodevelopmental character of psychosis. A prime candidate to achieve this might be neurocognitive intervention showing positive effect on cognition and functioning in patients suffering from SZ [7]. Further studies should investigate if the suggested clusters are shared between different phenotypes, particularly affective psychosis, and if common transdiagnostic pathways can be found for patients with cognitive impairments.

## Funding and disclosure

This work was supported in analysis and writing of the manuscript by the European Union-FP7 project PRONIA (“Personalized Prognostic Tools for Early Psychosis Management”, grant number 602152). JW was partly supported by the NARSAD Young Investigator Award of LK through the Brain and Behavior Research Foundation (grant number 28474). NK, JK and RKRA are currently honorary speakers for Otsuka/Lundbeck. RU achieved grants from Medical Research Council, grants from the National Institute for Health Research, and personal fees from Sunovion. The remaining authors including members of the PRONIA consortium have nothing to disclose.

## Supplementary information

Supplementary text

Supplementary figures and tables

Table S1

Table S2

Table S4

## Data Availability

The data models that support the findings of this study are available on request from the corresponding author [LK-I and NK]. The data are not publicly available due to ethical restrictions.
