# Supplementary material for: Cognitive subtypes in recent onset psychosis: distinct neurobiological fingerprints?
Source: Neuropsychopharmacology. 2021 Mar 15;46(8):1475–83. doi: 10.1038/s41386-021-00963-1 (PMC8209013; doi:10.1038/s41386-021-00963-1)
Supplement: Supplementary file 1 — Supplementary text [file 41386_2021_963_MOESM1_ESM.docx]

**Supplementary Material**

**General inclusion criteria**

Further general inclusion criteria were sufficient knowledge of the German or English language and capacity to provide informed consent. We tested participants that were either native speaker in English or German or bilingual participants with English or German as preferred language. Exclusion criteria for all subjects were the following: (1) No history of neurological disease, head trauma with loss of consciousness (> 5minutes), alcoholism or polytoxicomania; (2) no intellectual disability tested using the Wechsler Intelligence Scale for Adults [1]; (3) no more than 90 days of antipsychotic treatment in the last year according to DGPPN guidelines; (4) no condition that violated the MRI safety requirements.

The PRONIA study was registered at the German Clinical Trials Register (DRKS00005042) and received ethical approval from each study site’s institutional board [2]. Adult study participants gave their written informed consent prior to clinical and neuroimaging assessment. Study participants below the age of 18 provided informed assent accompanied by the informed consent of their caregivers. Consent was obtained according to the declaration of Helsinki.

**Exclusion of study participants and neurocognitive variables**

To ensure data quality we excluded patients with large amount of missing data (ROP: > 25%, N_ROP_=10; N_HC_=3). One patient of the discovery sample performed worse than 3 standard deviations below group average across several neurocognitive tasks and was excluded as an outlier. One study site (University of Birmingham) was excluded from the current analysis due to a small number of participants as compared to other study sites (ROP: N=2; HC: N=3). One study site (University of Turku) was excluded from the current analysis due to systematic missings in the Rey auditory verbal learning test (RAVLT). The procedure resulted in a data set of 108 ROP patients and 195 HC for the discovery sample and 53 ROP patients for the independent validation sample (table 1, figure S8).

In order to reduce the dimensionality in our discovery sample, we excluded neurocognitive variables with low variance (N=64), variables with task-inherent missing values (>15% missing; N=14) (table S2). The validation sample followed this variable selection strategy.

**Preprocessing of neurocognitive data: regression of covariates**

All selected neurocognitive variables were used. Missing values were imputed by the variable median, separately for ROP (~ 1.5%) and HC (< 0.5%) groups.

We regressed out confounding effects using a three-step procedure: (1) For each neuropsychological variable we built a multi-factorial regression model with sex, age, site and years of education as predictors based on the HC data set; (2) We applied this HC neuropsychological model to the corresponding neuropsychological variable of the ROP data set; (3) We took the residual values of this model for further analysis. This procedure was established in order to preserve effects of illness that interact with the predictors [3], to obtain a PRONIA sample specific standardization and to account for differences across study sites.

**Preprocessing of neurocognitive data: principal component analysis (PCA)**

The dimensionality of the cluster features was reduced by applying principal component analyses (PCA) on each group of the scaled neuropsychological variables associated with a certain cognitive domain (table S1). For each PCA the first principal component was retained, resulting in eight variables representing the performance in each of the cognitive domains (visual memory, social cognition, working memory, processing speed, verbal memory, executive functioning, attention and salience; figure S1). Prior to clustering analysis, all retained PCA components were scaled (variable mean subtracted from individual value divided by the variables’ standard deviation).

We projected the neuropsychological variables of the HC group and the validation sample into the PCA space of the discovery sample to ensure that PCA components were comparable across data sets. Therefore, we multiplied the new data matrices with the PCA weights associated with each principal component derived from the discovery sample.

We correlated each raw variable assigned to a certain domain with the corresponding PCA scale and determined directionality by majority vote. In the ROP and HC sample high PCA scores for social cognition, verbal memory, salience and executive functioning represent high performance and high PCA scores for working memory, attention, visual memory and processing speed represent low performance. In the ROP validation sample high PCA scores for social cognition, working memory, verbal memory and salience represent high performance and PCA scores for attention, visual memory, processing speed and executive functioning represent low performance.

**Clustering and cluster stability assessment using resampling**

A K-means clustering algorithm [4] was applied to the preprocessed neurocognitive variables using Euclidean distance. This algorithm partitions the data set into a pre-defined number (k) of clusters by minimizing the total within-cluster distance of observations to the cluster-specific centroid [5]. The number of clusters was determined by the maximum of the Calinski-Harabasz Index [6] and average silhouette width [7] over a cluster range from 2 to 10 (figure S2). Higher values represent a better ratio of within-cluster cohesion and between-cluster separation within both measures [8].

Two independent resampling strategies were followed to assess cluster stability [9]. The ‘subset’-method required to repetitively select a proportion of 50% of the total data set observations and the ‘noise’-method to repetitively replace 5% of the observations in the data set with noise. Both strategies were repeated for 50 times and Jaccard similarity [10] for each cluster solution across all resamples was calculated (figure S3). A Jaccard similarity value of below 0.6 is regarded as a dissolved, i.e. unstable, cluster solution, values between 0.6 and 0.75 indicate patterns in the data and values above 0.85 are considered as highly stable [9].

**Statistical analysis of demographic, clinical and neuropsychological effects between clusters and HC**

Demographic, clinical and neuropsychological characteristics of the obtained ROP subgroups and the HC sample were compared by applying one-way permutation and chi-squared tests using the ‘coin’- and the ‘rcompanion’-package [11,12]. We corrected all resulting p values within a certain contrast, i.e. impaired vs spared, impaired vs HC, spared vs HC and overall, separately, applying the Benjamini- Hochberg false discovery rate (FDR) method [13]. Pairwise group comparisons were only calculated in case of a significant (p < 0.05) overall group comparison.

**Post-hoc clustering analysis on premorbid IQ**

Effects for premorbid verbal and non-verbal intelligence comparing the impaired and spared cluster were obtained. In order to exclude that clusters were exclusively driven by premorbid intelligence we conducted another cluster analysis based solely on the premorbid verbal and non-verbal IQ variable in the data set with parameter specifications identical to the main analysis.

The most stable cluster solution split the data set into two clusters of 49 and 59 ROP patients. Subgroup assignments showed an overlap of ~ 74% indicating that not all of the variance obtained in the analysis can be explained by differences in premorbid intelligence. It is important to note that verbal and non-verbal Intelligence scores of the WAIS correlate with neuropsychological performance with on average r = 0.27 (r_min_ = 0.03; r_max_ = 0.44) (figure S7).

**Image preprocessing pipeline**

Prior to the machine learning analysis, imaging data was preprocessed using the open-source CAT12 toolbox (version >r1200; http://dbm.neuro.uni-jena.de/cat12/), an extension of the SPM12 software (Wellcome Department of Cognitive Neurology, London, UK; <http://www.fil.ion.ucl.ac.uk/spm/software/spm12/>). In accordance with previous studies [2] and the CAT12 manual ([www.neuro.uni-jena.de/cat12/CAT12-Manual.pdf](http://www.neuro.uni-jena.de/cat12/CAT12-Manual.pdf)) preprocessing followed the following steps: (1) denoising based on Spatially Adaptive Non-Local Means (SANLM) filtering [14]; (2) adjusting the images for white matter inhomogeneities and varying GM intensities using Local Adaptive Segmentation (LAS); (3) segmentation across cortical and subcortical structures by means of an Adaptive Maximum A Posteriori (AMAP) segmentation technique [15]; (4) additional denoising of the AMAP segmentation estimation using a Markov Random Field approach [15]; (5) application of a partial volume segmentation algorithm to model different tissue intensities (GM, white matter, cerebrospinal fluid) in the AMAP-generated tissue segments; (6) coregistration of the images to a MNI-template generated from the MRI data of 555 healthy controls in the IXI database (<http://www.braindevelopment.org>) and application of a Gaussian kernel 10 mm smoothing. Images were quality controlled using the CAT12 toolbox by correlating brain images among each other to check for signal homogeneity and by visual inspection.

**Machine learning pipeline: Nested cross-validation framework**

The applied machine learning pipeline encompassed the following steps:

1. For each 'fold-permutation-parameter’-combination, classification models were generated on the inner (CV1) training sample and evaluated on the held out inner test sample.
2. An optimum parameter setting was selected by majority vote on the balanced accuracies (BAC) of the generated models.
3. Subsequently, the whole inner loop sample (training, test) was used for retraining on the previously selected optimum parameter combination.
4. The resulting model was applied to the held-out outer (CV2) test sample and the final model performance was assessed.

This procedure was followed through all ‘fold-permutation’-combinations of the outer fold to (1) minimize the probability of overfitting, (2) evaluate the generalizability to ‘unseen’ data and (3) enable the optimization of model parameters. A comprehensive description of the nested cross-validation scheme can be found elsewhere [2].

**Machine learning pipeline: Estimation of voxel reliability across study sites using a G coefficient map**

Using generalization theory [16,17] a G coefficient map was calculated to estimate the reliability of grey matter (GM) voxels across study sites based on the data of HC traveling subjects (N=6), i.e. subjects being scanned at each MRI acquisition site [2]. Using the G coefficient map unreliable GM voxels were masked out within the inner loop of the machine learning framework prior to the application of the classification algorithm in order to avoid biases due to site-specific scanner properties and MRI protocols.

**Machine learning pipeline: Permutation testing of the classification model**

The BAC provides a general measure of the performance of the observed classification model. However, to obtain an estimate of single grey matter voxel contribution to the classification performance, the computation of permutation tests is recommended [18]. Therefore, classification labels (‘impaired’, ‘spared’, ‘HC’) were randomly shuffled (N=1000) and classification models were retrained on those label assignments, using model parameters and cross-validation identical to the observed model. The classification performance of the observed model is tested against the resulting null distribution of classification performances of the generated ‘random’ models. Significance is represented by the frequency of cases where the random model performs equal or better than the observed model divided by the total number of random models. A p-value (alpha < 0.5) can be derived [19].

**Machine learning pipeline: Visualisation of discriminative GM volume pattern**

To assess the reliability of voxels contributing to the classification performance of the winning model we inspected the cross-validation ratio. The cross-validation ratio represents the mean CV2 weight of all CV2 folds and permutations for a given voxel of the winning model divided by the CV1 standard error. More extreme weight values indicate a stronger contribution to the classification performance. The direction of the weight value (positive or negative) indicates whether a given voxel is predictive of one group or the other [2].

**Post-hoc correlation analysis between decision scores and relevant variables**

In order to relate our neuroanatomical classification model to clinical and functional variables not used for classification, we calculated Pearson correlation coefficients between the decision scores of the significant ‘impaired subgroup vs HC’ classification model and several clinical measures and premorbid intelligence scores.

We observed significant correlations between support vector machine decision scores for the ‘impaired subgroup vs HC’ classification model and non-verbal premorbid Intelligence (r_pearson_= -0.26, p < 0.001), GF (r_pearson_= -0.19, p < 0.01) and GAF (r_pearson_= -0.19, p < 0.01) (figure S6, table S3).

Further, to exclude that decision scores were driven by scanner differences between study sites we calculated an analysis of variance (ANOVA) with decision scores as dependent and study site as independent variable for the ‘impaired subgroup vs HC’ model. This model showed no significant effect of study site on the decision scores (F(1, 234) = 0.68, p = 0.412).

**Members of the PRONIA consortium**

Department of Psychiatry and Psychotherapy, Ludwig-Maximilian-University, Munich, Bavaria, Germany: Anne Erkens, Eva Gussmann, BSc, Shalaila Haas, MSc, Alkomiet Hasan, MD, Claudius Hoff, MD, Ifrah Khanyaree, BSc, Aylin Melo, BSc, Susanna Muckenhuber-Sternbauer, MD, Janis Köhler, Ömer Öztürk, MD, David Popovic, MD, Adrian Rangnick, BSc, Sebastian von Saldern, MD, Rachele Sanfelici, MSc, Moritz Spangemacher, Ana Tupac, MSc, Maria Fernanda Urquijo, MSc, Johanna Weiske, MSc, and Antonia Wosgien.

University of Cologne, North Rhineland–Westphalia, Germany: Dennis Hedderich, MD, Karsten Blume, Mauro Seves, MSc, Nathalie Kaiser, MSc, Thorsten Lichtenstein, MD, Christiane Woopen, MD, Linda Betz, BSc and Nora Penzel, MSc.

Psychiatric University Hospital, University of Basel, Basel, Switzerland: Christina Andreou, MD, PhD, Laura Egloff, PhD, Fabienne Harrisberger, PhD, Claudia Lenz, PhD, Letizia Leanza, MSc, Amatya Mackintosh, MSc, Renata Smieskova, PhD, Erich Studerus, PhD, Anna Walter, MD, and Sonja Widmayer, MSc.

Institute of Mental Health, University of Birmingham, Birmingham, United Kingdom: Chris Day, BSc, Sian Lowri Griffiths, PhD, Mariam Iqbal, BSc, Mirabel Pelton, MSc, Pavan Mallikarjun, MBBS, DPM, MRCPsych, PhD, Alexandra Stainton, MSci, and Ashleigh Lin, PhD.

Department of Psychiatry, University of Turku, Turku, Finland: Alexander Denissoff, MD, Anu Ellilä, RN, Tiina From, MSc, Markus Heinimaa, MD, PhD, Tuula Ilonen, PhD, Päivi Jalo, RN, Heikki Laurikainen, MD, Maarit Lehtinen, RN, Antti Luutonen, BA, Akseli Mäkela, BA, Janina Paju, MSc, Henri Pesonen, PhD, Reetta-Liina Armio (Säilä), MD, Elina Sormunen, MD, Anna Toivonen, MSc, and Otto Turtonen, MD.

General Electric Global Research Inc, Munich, Germany: Ana Beatriz Solana, PhD, Manuela Abraham, MBA, Nicolas Hehn, PhD, and Timo Schirmer, PhD.

Workgroup of Paolo Brambilla, MD, PhD University of Milan, Milan, Italy: Department of Neuroscience and Mental Health, Fondazione IRCCS Ca' Granda Ospedale Maggiore Policlinico, University of Milan, Milan, Italy: Carlo Altamura, MD, Marika Belleri, PsychD, Francesca Bottinelli, PsychD, Adele Ferro PsychD, PhD, and Marta Re, PhD. Programma2000, Niguarda Hospital, Milan: Emiliano Monzani, MD, Mauro Percudani, MD, and Maurizio Sberna, MD. San Paolo Hospital, Milan: Armando D’Agostino, MD, and Lorenzo Del Fabro, MD. Villa San Benedetto Menni, Albese con Cassano: Giampaolo Perna, MD, Maria Nobile MD, PhD, and Alessandra Alciati, MD.

Workgroup of Paolo Brambilla, University of Udine, Udine, Italy: Department of Medical Area, University of Udine: Matteo Balestrieri, MD, Carolina Bonivento, PsychD, PhD, Giuseppe Cabras, PhD, and Franco Fabbro, MD, PhD. IRCCS Scientific Institute “E. Medea”, Polo FVG, Udine: Marco Garzitto, PsychD, PhD and Sara Piccin, PsychD, PhD.

**References**

1. Wechsler D. Manual for the Wechsler adult intelligence scale. Psychological Corp; 1955.
2. Koutsouleris N, Kambeitz-Ilankovic L, Ruhrmann S, Rosen M, Ruef A, Dwyer DB, et al. Prediction models of functional outcomes for individuals in the clinical high-risk state for psychosis or with recent-onset depression: a multimodal, multisite machine learning analysis. JAMA psychiatry. 2018;75(11):1156–72.
3. Koutsouleris N, Meisenzahl EM, Borgwardt S, Riecher-Rössler A, Frodl T, Kambeitz J, et al. Individualized differential diagnosis of schizophrenia and mood disorders using neuroanatomical biomarkers. Brain. 2015;138(7):2059–73.
4. Lloyd S. Least squares quantization in PCM. IEEE Trans Inf Theory. 1982;28(2):129–37.
5. James G, Witten D, Hastie T, Tibshirani R. An introduction to statistical learning. Vol. 112. Springer; 2013.
6. Caliński T, Harabasz J. A dendrite method for cluster analysis. Commun Stat Theory Methods. 1974;3(1):1–27.
7. Rousseeuw PJ. Silhouettes: a graphical aid to the interpretation and validation of cluster analysis. J Comput Appl Math. 1987;20:53–65.
8. Zumel N, Mount J. Practical data science with R. Manning Publications Co.; 2014.
9. Hennig C. Cluster-wise assessment of cluster stability. Comput Stat Data Anal. 2007;52(1):258–71.
10. Jaccard P. Nouvelles recherches sur la distribution florale. Bull Soc Vaud Sci Nat. 1908;44:223–70.
11. Hothorn T, Hornik K, Van De Wiel MA, Zeileis A. A lego system for conditional inference. Am Stat. 2006;60(3):257–63.
12. Mangiafico S. rcompanion: Functions to support extension education program evaluation. R package version. 2017;1(0).
13. Benjamini Y, Hochberg Y. Controlling the false discovery rate: a practical and powerful approach to multiple testing. J R Stat Soc Series B Stat Methodol. 1995;57(1):289–300.
14. Manjon JV, Tohka J, García‐Martí G, Carbonell‐Caballero J, Lull JJ, Martí‐Bonmatí L, et al. Robust MRI brain tissue parameter estimation by multistage outlier rejection. Magn Reson Med. 2008;59(4):866–73.
15. Rajapakse JC, Giedd JN, Rapoport JL. Statistical approach to segmentation of single-channel cerebral MR images. IEEE Trans Med Imaging. 1997;16(2):176–86.
16. Brennan RL. Generalizability theory and classical test theory. Appl Meas Educ. 2010;24(1):1–21.
17. Mushquash C, O’Connor BP. SPSS and SAS programs for generalizability theory analyses. Behav Res Methods. 2006;38(3):542–7.
18. Gaonkar B, Davatzikos C. Analytic estimation of statistical significance maps for support vector machine based multi-variate image analysis and classification. Neuroimage. 2013;78:270–83.
19. Golland P, Fischl B. Permutation tests for classification: towards statistical significance in image-based studies. In Springer; 2003. p. 330–41.
20. Osterrieth P. The test of copying a complex figure: A contribution to the study of perception and memory. Arch Psychol. 1944;30:206–356.
21. Shin M-S, Park S-Y, Park S-R, Seol S-H, Kwon JS. Clinical and empirical applications of the Rey–Osterrieth complex figure test. Nat Protoc. 2006;1(2):892.
22. Nuechterlein KH, Green MF, Kern RS, Baade LE, Barch DM, Cohen JD, et al. The MATRICS Consensus Cognitive Battery, part 1: test selection, reliability, and validity. Am J Psychiatry. 2008;165(2):203–13.
23. Baddeley A. The episodic buffer: a new component of working memory? Trends Cogn Sci. 2000;4(11):417–23.
24. Cavaco S, Gonçalves A, Pinto C, Almeida E, Gomes F, Moreira I, et al. Semantic fluency and phonemic fluency: regression-based norms for the Portuguese population. Arch Clin Neuropsychol. 2013;28(3):262–71.
25. Greenaway MC, Smith GE, Tangalos EG, Geda YE, Ivnik RJ. Mayo older americans normative studies: factor analysis of an expanded neuropsychological battery. Clin Neuropsychol. 2009;23(1):7–20.
26. Arbuthnott K, Frank J. Trail making test, part B as a measure of executive control: validation using a set-switching paradigm. J Clin Exp Neuropsychol. 2000;22(4):518–28.
27. Ross TP, Hanouskova E, Giarla K, Calhoun E, Tucker M. The reliability and validity of the self-ordered pointing task. Arch Clin Neuropsychol. 2007;22(4):449–58.
28. Petrides M, Milner B. Deficits on subject-ordered tasks after frontal-and temporal-lobe lesions in man. Neuropsychologia. 1982;20(3):249–62.
29. Schmidt K, Roiser JP. Assessing the construct validity of aberrant salience. Front Behav Neurosci. 2009;3:58.
