# Supplementary material for: Cognitive subtypes in recent onset psychosis: distinct neurobiological fingerprints?
Source: Neuropsychopharmacology. 2021 Mar 15;46(8):1475–83. doi: 10.1038/s41386-021-00963-1 (PMC8209013; doi:10.1038/s41386-021-00963-1)
Supplement: Supplementary file 2 — Supplementary figures and tables [file 41386_2021_963_MOESM2_ESM.docx]

**Supplementary figures**


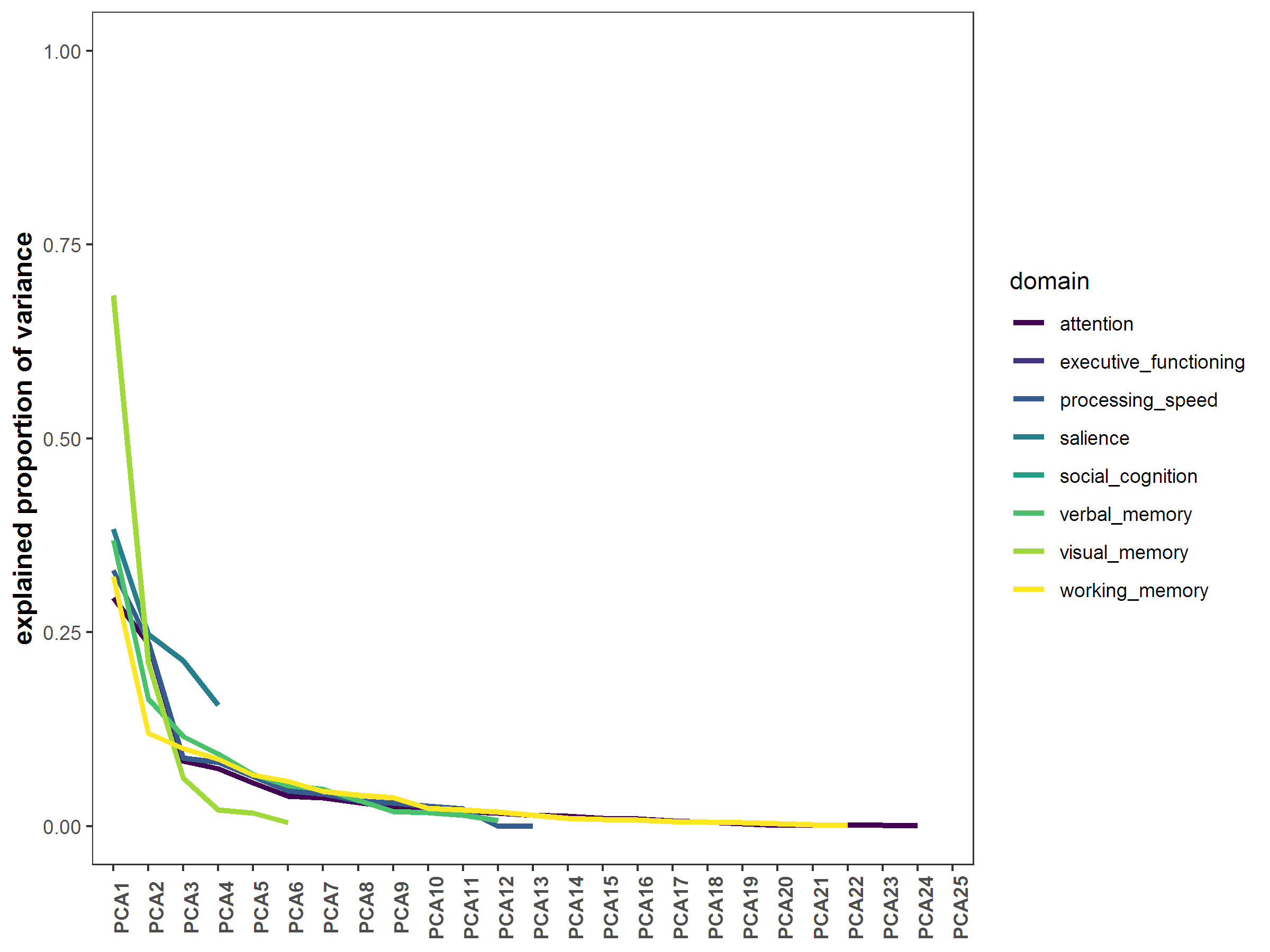


**Figure S1.** Explained proportions of variance of principal component analyses (PCA) calculated on cognitive domains of the discovery recent onset psychosis (ROP) sample.


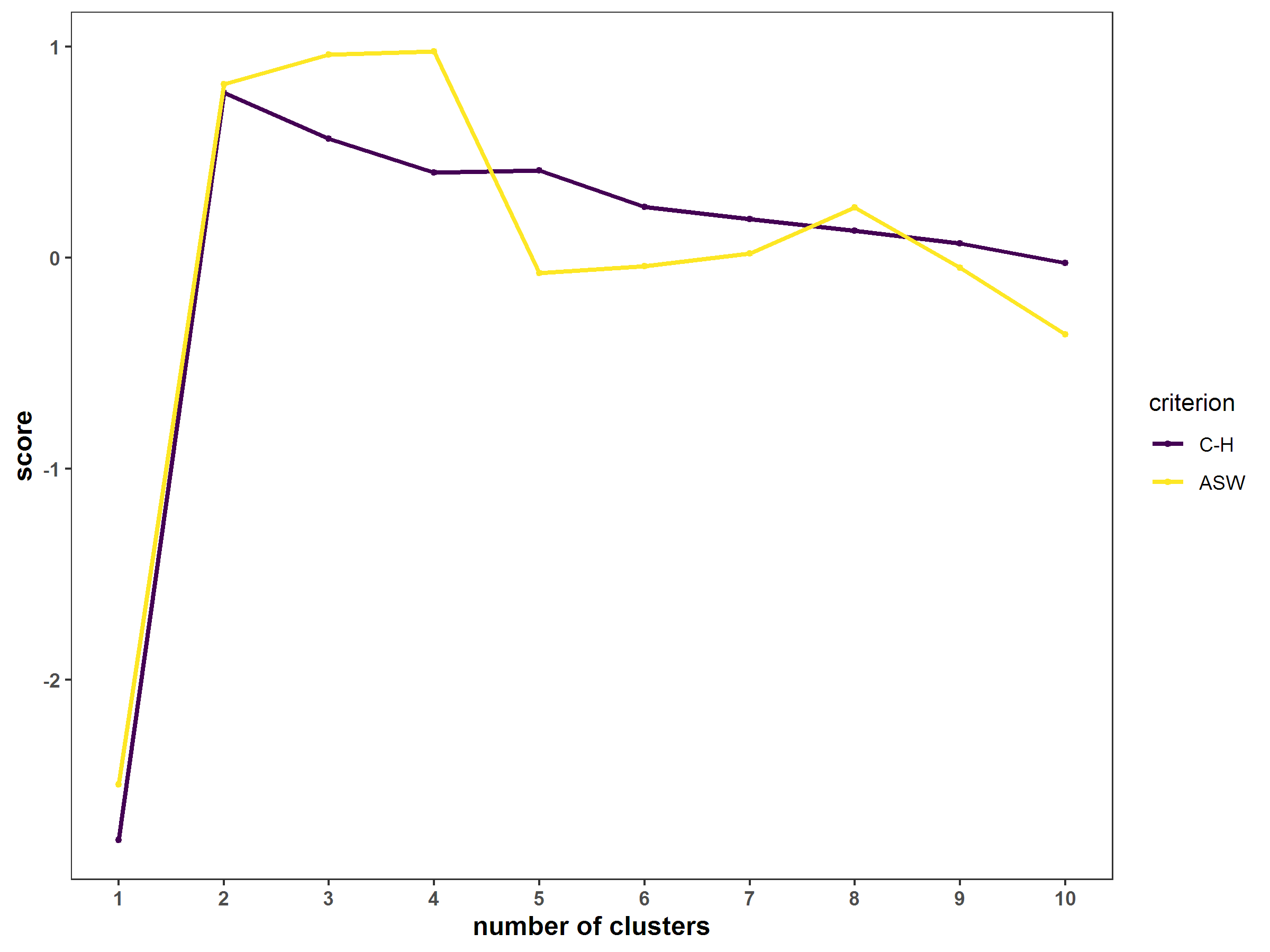


**Figure S2.** Calinski-Harabasz and average silhouette width score for a cluster range of 2 to 10 clusters using a K-means clustering algorithm. For the analyses of cluster characteristics, a two-cluster solution was chosen.


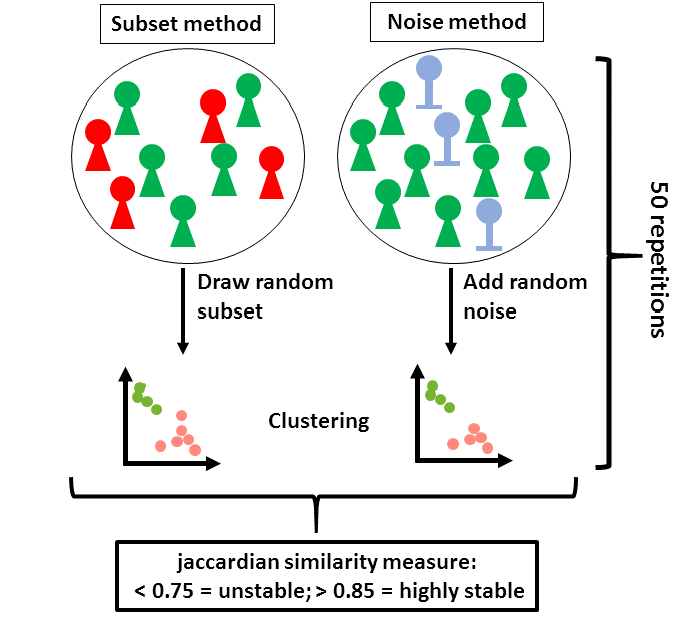


**Figure S3**. Resampling scheme for cluster-wise stability assessment according to [9]. Two methods were applied: ‘subset’ and ‘noise’. Data was resampled for 50 times and similarity across the obtained cluster solutions was assessed by the Jaccard similarity index [10].


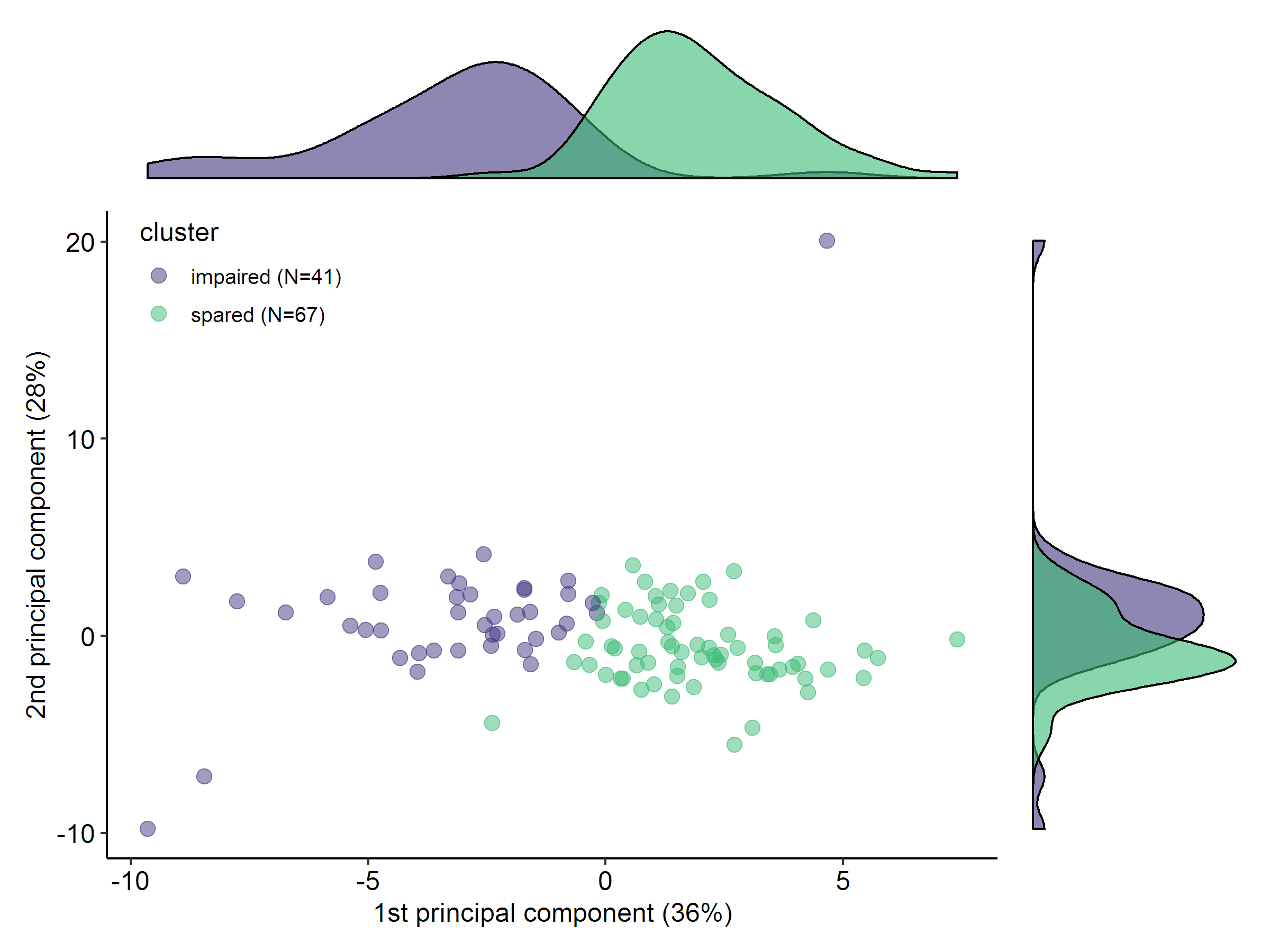


**Figure S4.** Two-cluster solution projected into the first and second principal component of the neuropsychological data space with smoothed histograms attached. Within the resampling approach, cluster-wise Jaccard similarity scores indicated highly stable clusters.


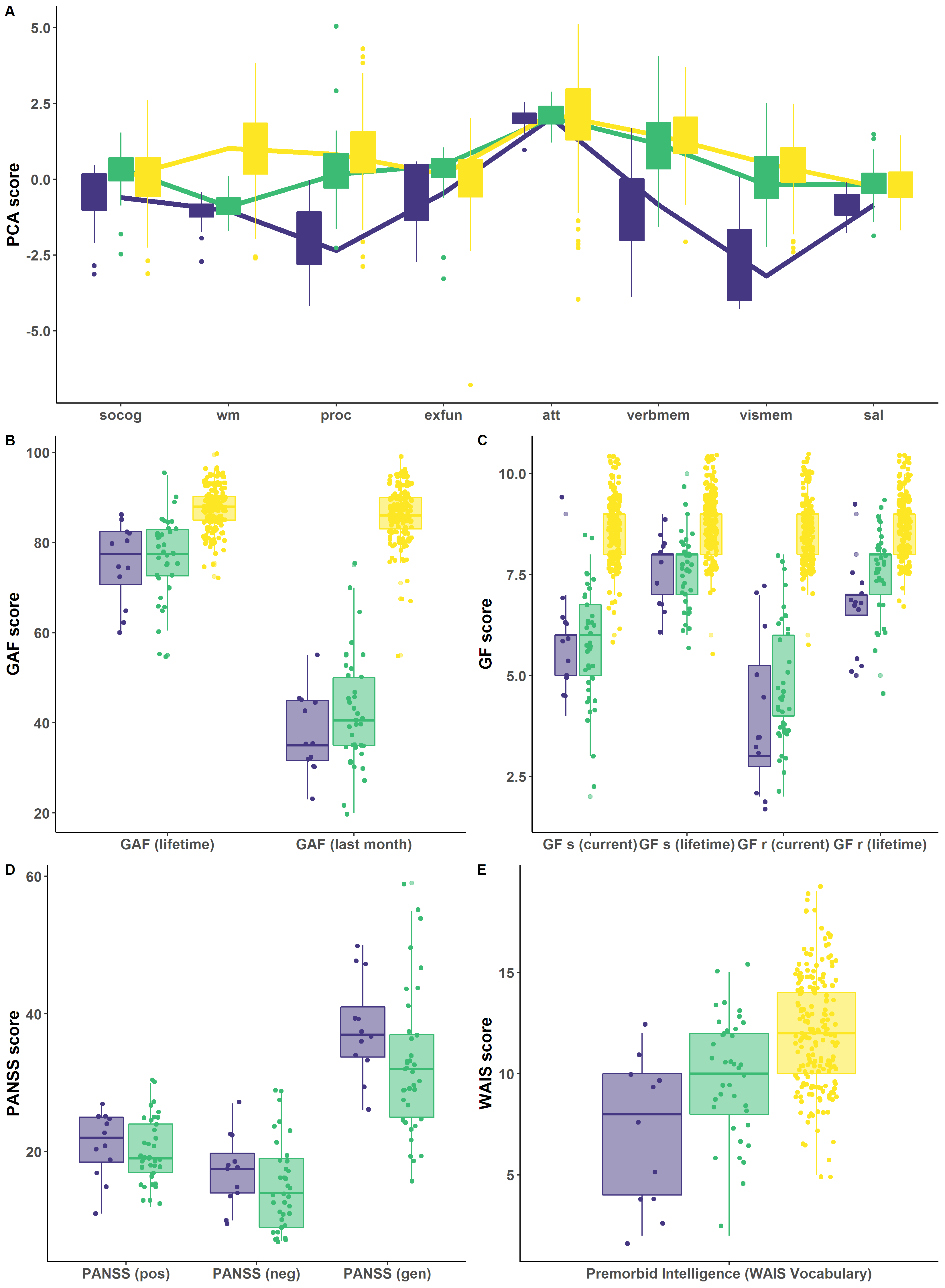


**Figure S5.** Neuropsychological and clinical differences between clusters and HC in the validation sample. Observations assigned to the impaired cluster (N=13) are depicted in ‘blue’, observations assigned to the spared cluster (N=40) are depicted in ‘green’ and healthy controls (HC; N=195) are depicted in yellow. Differences between groups are shown regarding (A) the neuropsychological PCA components, (B) the General Assessment of Functioning score (GAF), (C) the General Functioning score (GF), (D) the Positive and Negative Syndrom Scale (PANSS) and (D) premorbid verbal intelligence. In section (A) high PCA scores represent high performance. PCA scales for cognitive domains where high PCA scores represent low performance, are inverted. Abbrev: socog = social cognition; wm = working memory; proc = processing speed; exfun = executive functioning; att = attention; verbmem = verbal memory; vismem = visual memory; sal = salience.


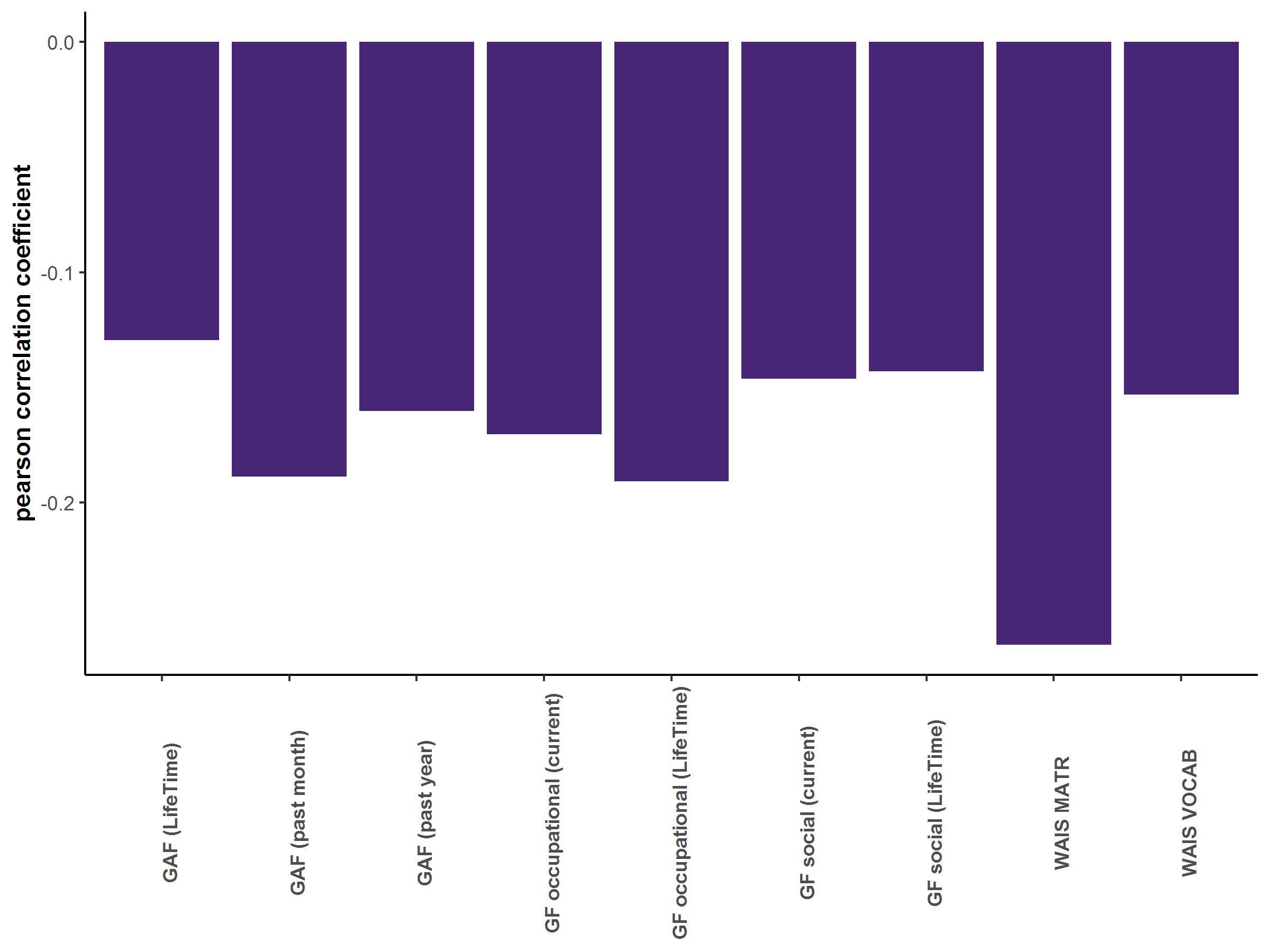


**Figure S6.** Pearson correlation coefficients calculated between the SVM decision scores of the ‘impaired subgroup vs HC’ classification model and clinical measures. A negative correlation coefficient indicates that high values in behavioral measures are associated with HC status (negative decision scores). All correlations are significantly different from 0 (p < 0.05).


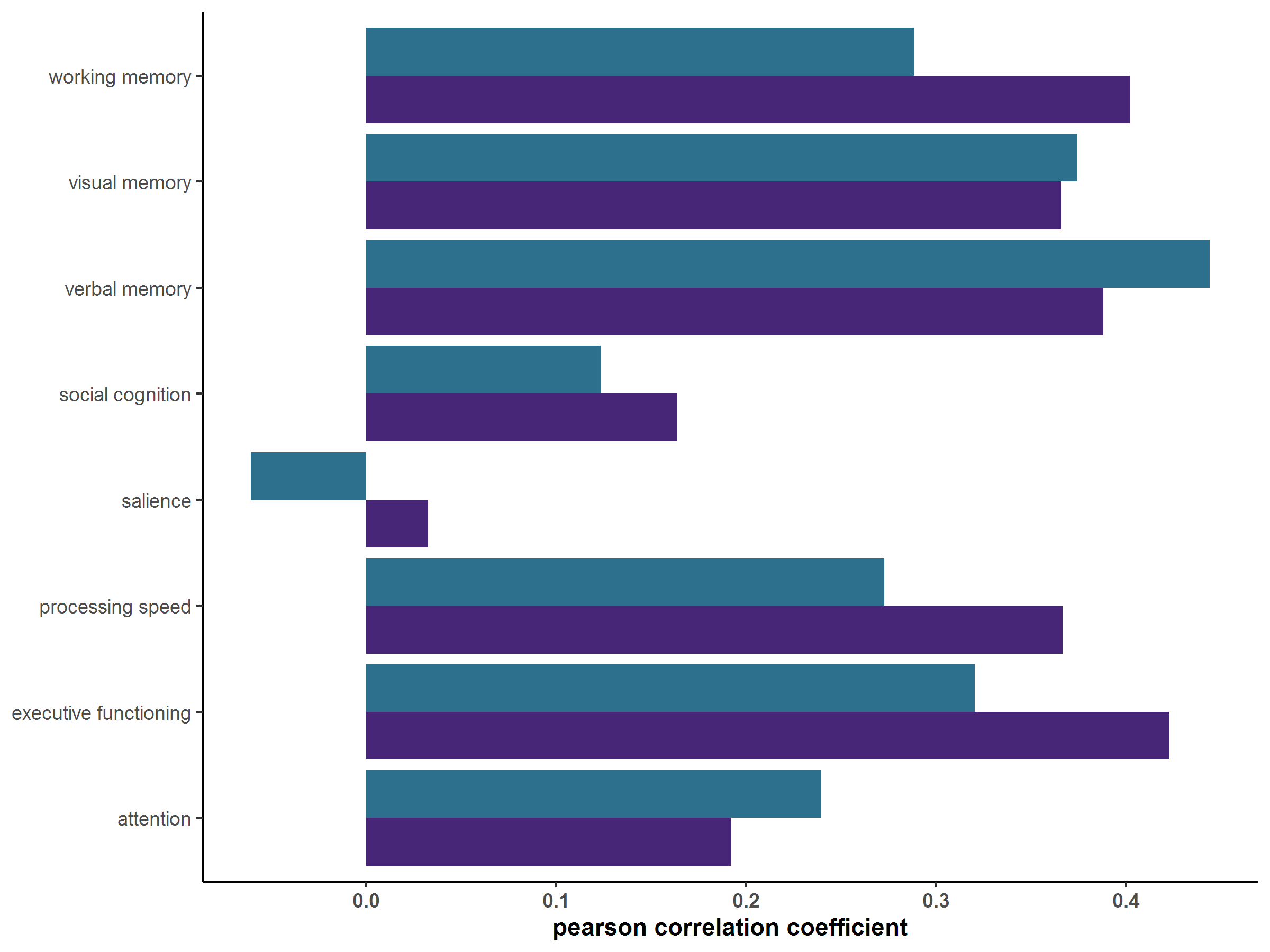


**Figure S7.** Pearson correlation coefficients between premorbid IQ scores and PCA scores of neuropsychological domains. Dark blue bars represent the matrices IQ subtest and light blue bars represent the vocabulary IQ subtest. High PCA scores represent high performance. PCA scales for cognitive domains where high PCA scores represent low performance, are inverted.


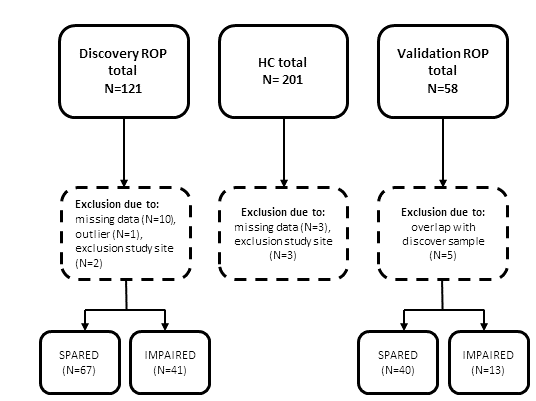


**Figure S8.** Flow chart of exclusion of cases. Cases were excluded due to the amount of missing data (> 25%), being outlier and showing overlap with the discovery sample. Further, several cases were excluded as they were recruited by a study site underrepresented in the data set. The analysis data sets consisted of 108, 195, 53 patients for ROP (discovery), HC and ROP (validation), respectively.

**Supplementary tables**

**Table S1.** **Neuropsychological tests used in the study and associated cognitive domains.**

Note. ^a^ revised version of the Hopkins’ Verbal Learning Test for the finish study site, ^b^ test not used for clustering analysis.

**Table S2. Included and excluded neuropsychological variables of the current study.**

**Table S3**. **Pearson correlation coefficients between the decision scores of the significant classification model (impaired cluster against HC) and several clinical measures and premorbid intelligence scores.**

Note. Significance: * p < 0.05, ** p < 0.01, *** p < 0.001.

**Table S4. Clinical characteristics of subgroups in discovery and validation sample.**

Note. Abbrev: HC = healthy control; GAF = General Assessment of Functioning; GF = General Functioning Score; WAIS = Wechsler Intelligence Score; PANSS = Positive and Negative Syndrome Scale; BDI = Becks Depression Inventory. Significance: * p < 0.05, ** p < 0.01, *** p < 0.001.
